# Supplementary figures and images for: Climate change increases threat to plant diversity in tropical forests of Central America and southern Mexico
Source: PLoS One. 2024 Feb 29;19(2):e0297840. doi: 10.1371/journal.pone.0297840 (PMC10903834; doi:10.1371/journal.pone.0297840)

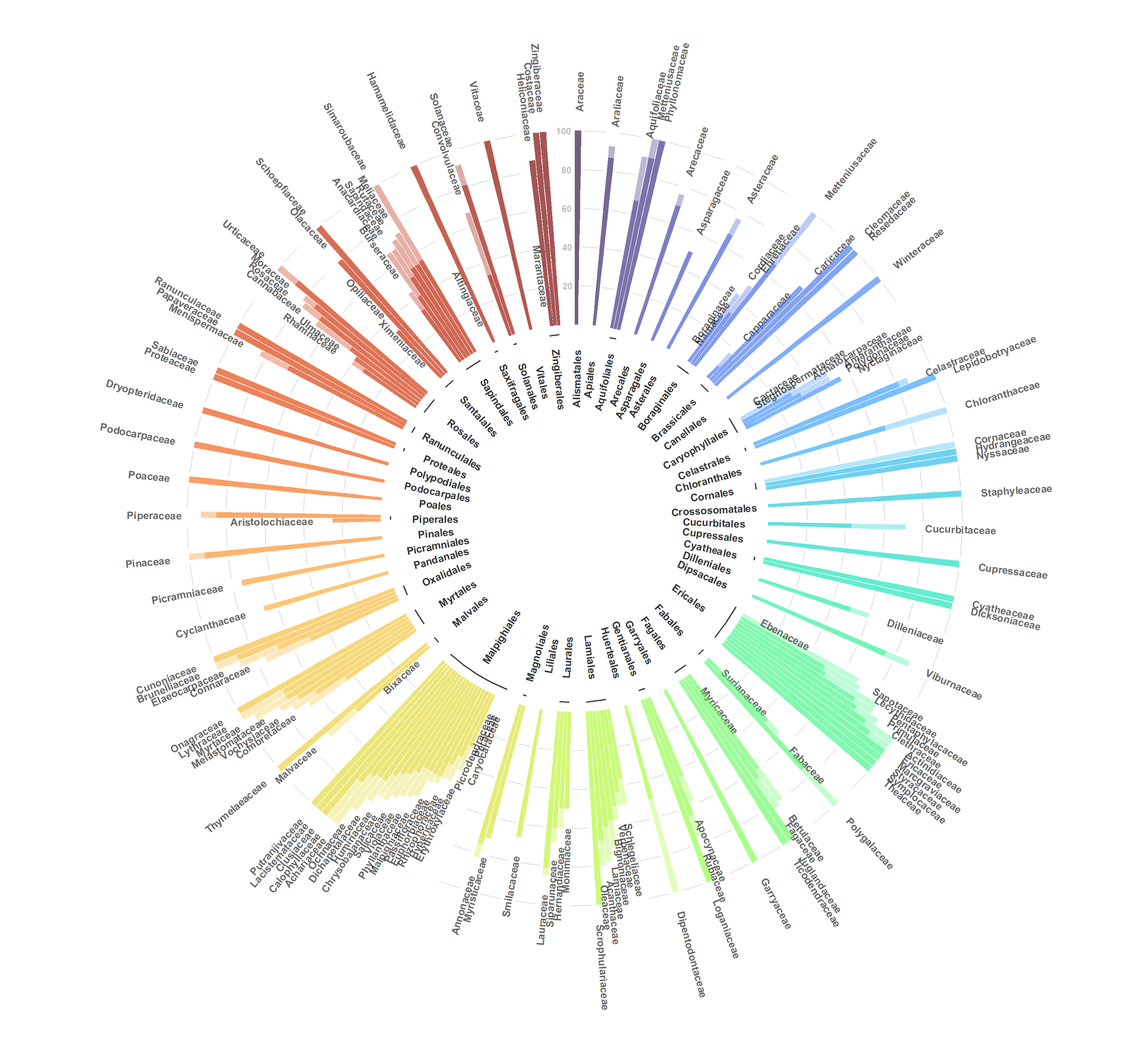

Supplement: S1 Fig — RCP4.5 (dark bars) and RCP8.5 (dark + light bars) under the assumption of limited dispersal. Different colors indicate different plant orders. (TIF) [file pone.0297840.s001.tif]
