# Supplementary material for: Climate change increases threat to plant diversity in tropical forests of Central America and southern Mexico
Source: PLoS One. 2024 Feb 29;19(2):e0297840. doi: 10.1371/journal.pone.0297840 (PMC10903834; doi:10.1371/journal.pone.0297840)

SUPPLEMENTARY MATERIAL. TABLE S1. Pairwise Pearson correlation analysis, showing r values and confidence intervals.

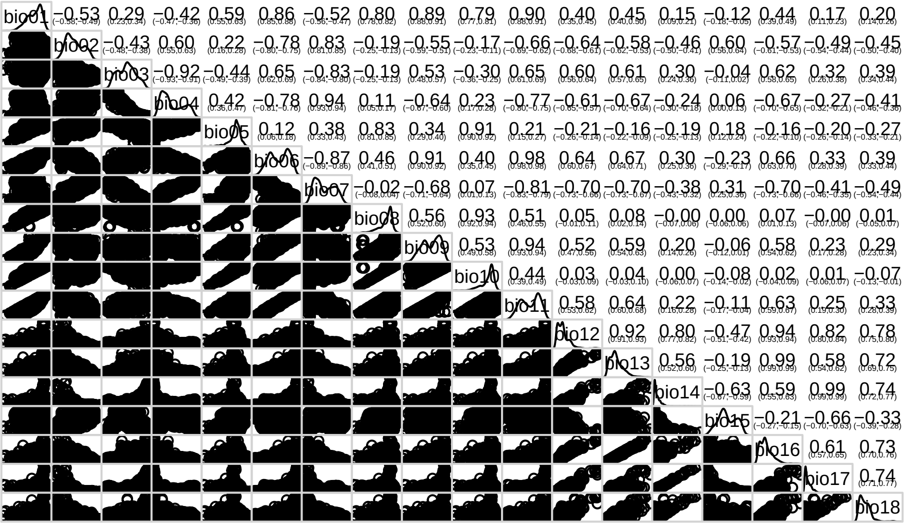

Supplement: S2 Table — Cells above the diagonal show the r value; cells below the diagonal show the density distribution. (PDF) [file pone.0297840.s003.pdf]
